# Supplementary material for: Single‐Atom Control of Arsenic Incorporation in Silicon for High‐Yield Artificial Lattice Fabrication
Source: Adv Mater. 2024 Mar 22;36(24):2312282. doi: 10.1002/adma.202312282 (PMC11475292; doi:10.1002/adma.202312282)
Supplement: Supplementary file 1 — Supporting Information [file ADMA-36-2312282-s001.pdf]

ADVANCED MATERIALS

Supporting Information

for *Adv. Mater.*, DOI 10.1002/adma.202312282

Single-Atom Control of Arsenic Incorporation in Silicon for High-Yield Artificial Lattice Fabrication

Taylor J. Z. Stock, Oliver Warschkow, Procopios C. Constantinou, David R. Bowler, Steven R. Schofield and Neil J. Curson**

Supporting Information

Single-Atom Control of Arsenic Incorporation in Silicon for High-Yield Artificial Lattice Fabrication

Taylor J. Z. Stock, Oliver Warschkow, Procopios C. Constantinou, David R. Bowler, Steven R. Schofield, Neil J. Curson**

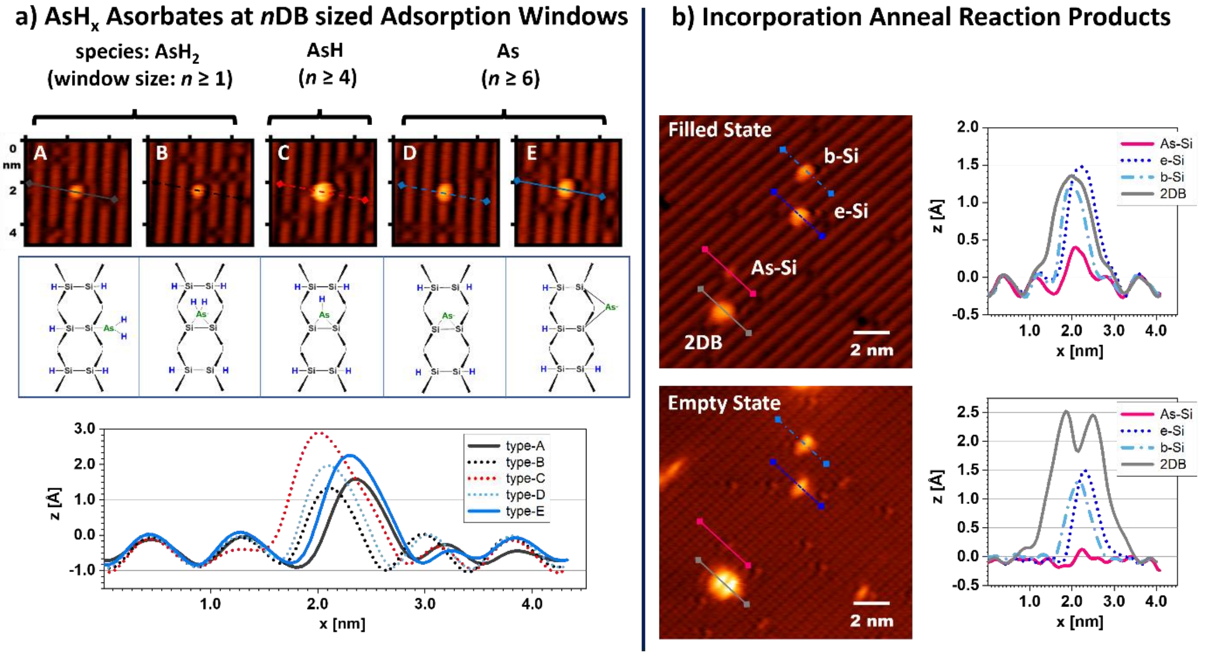

Figure S1. AsH$_x$ Adsorption Species and Thermal Anneal Reaction Products: When 1 to 6 DB sized hydrogen resist adsorption windows are exposed to AsH_3 gas, a variety of AsH$_x$ adsorbate features are detected, dependent on the window size. Panel a) shows filled state STM images and extracted line profiles of the five regularly observed adsorbate feature types, labelled A-E. These five species are divided into three categories based on the assumed degree of dissociation, corresponding to AsH$_x$, where x = 0, 1, or 2. Possible structure diagrams are provided below the corresponding images for each feature type. Species pairs A/B and D/E can only be distinguished in STM images displaying atomic resolution. When differentiation of these pairs is not possible, the full adsorbate set collapses into three species designated as A/B (AsH_2), C (AsH), and D/E (As) When annealed above 350 °C, the five AsH$_x$ adsorbates can produce a variety of reaction products. Panel b) shows filled and empty state STM images and extracted line profiles of four possible products: adsorbed silicon monomers e-Si (end-bridge) and b-Si (dimer bridge), arsenic-silicon heterodimer (As-Si), and silicon dangling bonds (2DB). The above identification of adsorbed reactants and products in STM images provides a key to assessing sequential images of H:Si(001)-(2×1) surfaces that have undergone all or some of the three-step process of lithography-dose-anneal. Imaging parameters: a) -2.0 V, 60 pA; b), c) ±2.0 V, 100 pA. False colour scales: a) -100..350 pm; b) -50..250 pm.

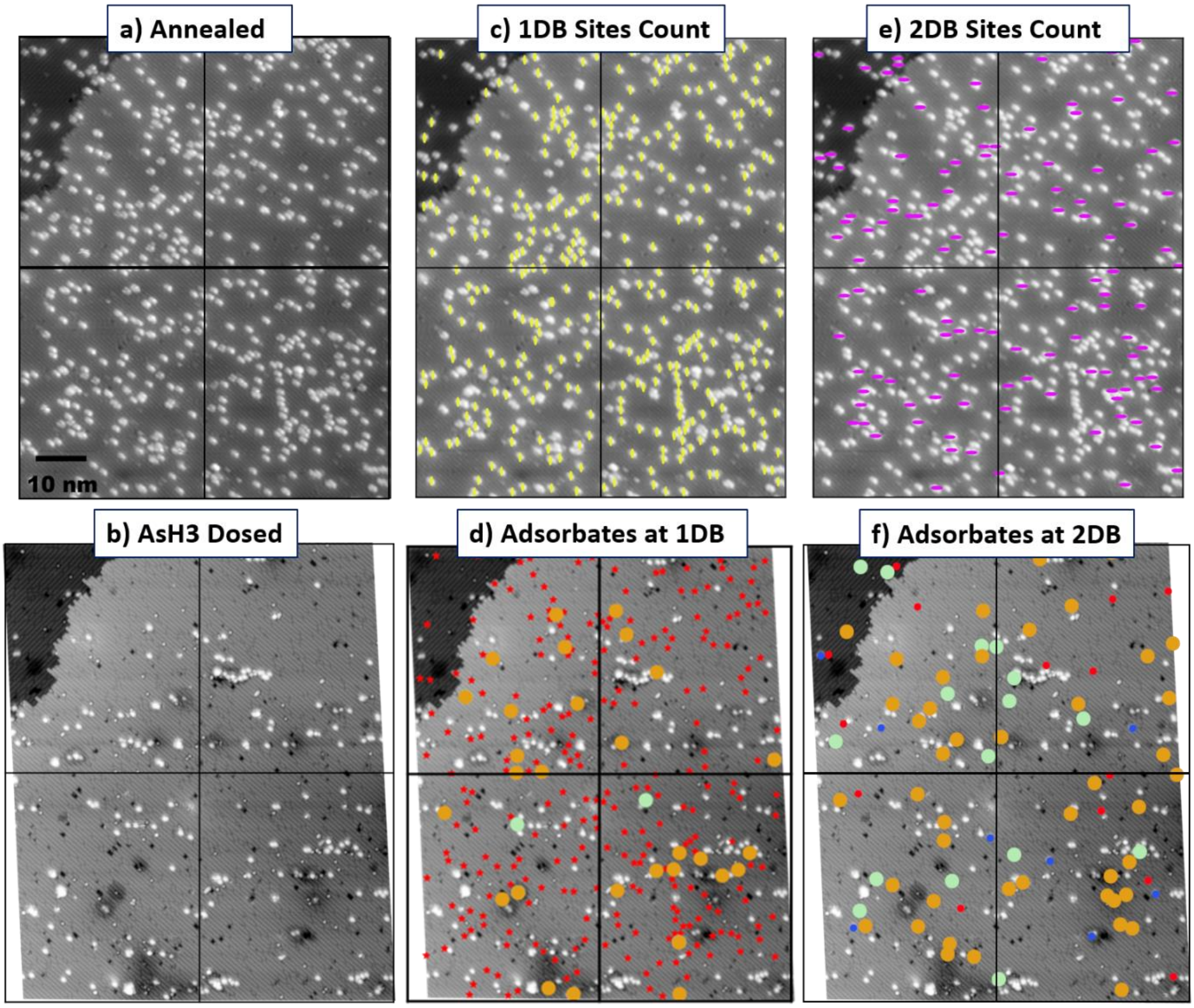

Figure S2. AsH_3 Adsorption Statistics at 1DB and 2DB Adsorption Sites: The same 100×80 nm area of an H:Si(001) surface is shown in panel a) following a ~375 °C anneal, and then in b) following a 3 L AsH_3 exposure. The anneal produces a high density of 1DB and 2DB sites, and the AsH_3 exposure converts these sites into AsH_3 related adsorbates. In panel c) all 1DB sites are marked with yellow vertical strikes, and in panel e) all 2DB sites are marked with purple horizontal strikes. In panels d) and f) the marked 1DB and 2DB sites, respectively, are followed through their conversion following AsH_3 adsorption. Adsorbate feature type-A are marked with a large brown circle and type-B with a large green circle. Hydrogen terminated sites are marked with a small red circle (or blue when a 2DB site is converted to a 1DB). Unexpected occasional adsorption of type-B features at 1DB sites is attributed to conversion of those 1DB sites to 2DB during the imaging process via spurious STM tip desorption (evidence of such tip desorption is found in the vertical strips of clustered adsorbates in the AsH_3 dosed image). The statistics of Table 1 and Figure 3i are generated by identifying and then counting the DB sites and adsorbates as illustrated here.

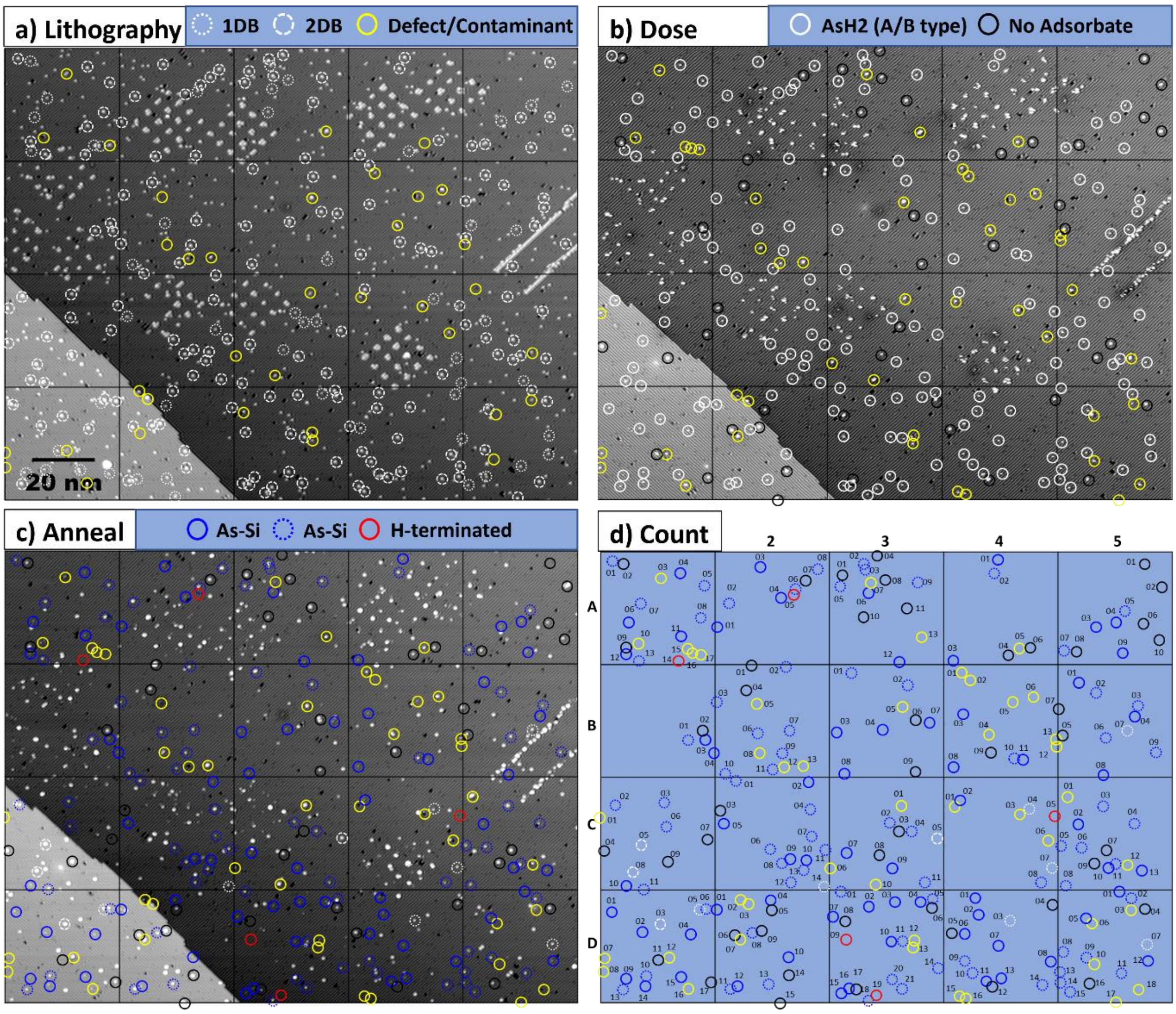

Figure S3. Arsenic Incorporation Statistics at 1DB and 2DB Adsorption Sites: The same 190 ×150 nm area of a H:Si(001) surface is shown following a) lithographic patterning, b) AsH_3 dosing (3 L), and c) thermal annealing (~350 °C) for dopant incorporation. In image (a), thermally generated 1DB and 2DB defects are marked with dotted or dashed white circles, respectively, and pre-existing defects/contaminants (including DB sites immediately next to defects/contaminants) are marked with solid yellow circles. In image (b) 1DB and 2DB sites are assessed for adsorption and marked with a solid white circle for a type A/B AsH_2 adsorbate, solid black circle for no adsorbate, or solid yellow circle as before (note: two A/B features immediately next to each other are counted as a defect). In image (c) the AsH_2 adsorbates are assessed for incorporation and marked with a solid blue circle for a As-Si heterodimer, dashed blue circle for ad-Si, dashed/dotted white circle for DBs as before, and a solid red circle for hydrogen termination. Finally, in panel d) all recorded features are counted. The statistics of Table 1 and Figure 3l are generated following this procedure.

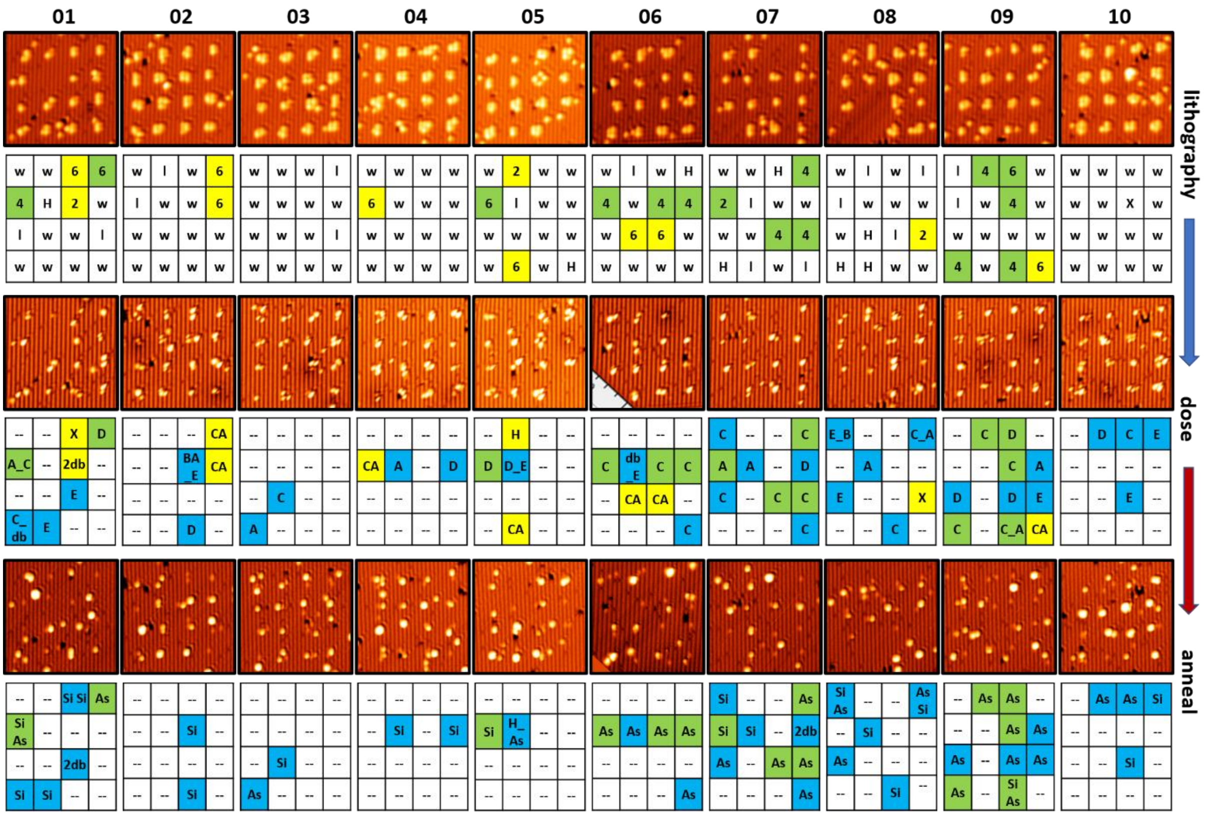

Figure S4. Adsorption and Incorporation Statistics at Lithographic Adsorption Windows: The 30 STM images above show ten 4×4 arrays patterned on a single atomic terrace of H:Si(001) shown in Figure S3. Each array is ~20×20 nm with a ~5 nm pitch. As indicated on the right-hand side of the image rows, all ten arrays (number at top of column) are followed through the three-step process of STM-HRL fabrication: lithographic patterning, arsine dosing, and thermal annealing for incorporation. At each process step, the array elements are assessed and results are tabulated in the grids immediately below each image; yellow grid cells are followed between process stages one and two, blue between stages two and three, and green are followed through all three stages. In the lithography stage, array elements are assessed for size and labelled "2", "4", or "6" indicating the number of DBs in a symmetric single-row lithographic window, or "l" or "w" indicating windows that are too long or too wide. In the dosing stage, all AsH_x adsorbates, including clusters of adsorbates, are tabulated according to feature type "A", "C", "D", or "E", or "X" for contaminant. Two adsorbates in the same array element separated by at least one dimer row are indicated by an underscore. Finally, in the anneal stage, any isolated single adsorbate feature from the dosing images (including those that are found at a "w" or "l" window) are assessed for incorporation. The reaction products are recorded as "As", "Si", "H", or "db" indicating As-Si heterodimer, ad-Si, hydrogen termination, or dangling bond(s). Statistics in Figures 3i and 3l are generated using this procedure to evaluate the images above as well as data shown in Figures 3a and 3e.
